# Role of Biliverdin Reductase A in the Regulation of Insulin Signaling in Metabolic and Neurodegenerative Diseases: An Update

**DOI:** 10.3390/ijms23105574

**Published:** 2022-05-16

**Authors:** Flavia Agata Cimini, Marzia Perluigi, Ilaria Barchetta, Maria Gisella Cavallo, Eugenio Barone

**Affiliations:** 1 Department of Experimental Medicine, Sapienza University of Rome, 00185 Rome, Italy; flaviaagata.cimini@uniroma1.it (F.A.C.); ilaria.barchetta@uniroma1.it (I.B.); 2 Department of Biochemical Sciences “A. Rossi-Fanelli”, Sapienza University of Rome, 00185 Rome, Italy; marzia.perluigi@uniroma1.it (M.P.); eugenio.barone@uniroma1.it (E.B.)

**Keywords:** biliverdin reductase A, obesity, type 2 diabetes, Alzheimer’s disease, dementia, insulin signaling, metabolic disorders, neurodegenerative diseases

## Abstract

Insulin signaling is a conserved pathway that orchestrates glucose and lipid metabolism, energy balance, and inflammation, and its dysregulation compromises the homeostasis of multiple systems. Insulin resistance is a shared hallmark of several metabolic diseases, including obesity, metabolic syndrome, and type 2 diabetes, and has been associated with cognitive decline during aging and dementia. Numerous mechanisms promoting the development of peripheral and central insulin resistance have been described, although most of them were not completely clarified. In the last decades, several studies have highlighted that biliverdin reductase-A (BVR-A), over its canonical role in the degradation of heme, acts as a regulator of insulin signaling. Evidence from human and animal studies show that BVR-A alterations are associated with the aberrant activation of insulin signaling, metabolic syndrome, liver steatosis, and visceral adipose tissue inflammation in obese and diabetic individuals. In addition, recent findings demonstrated that reduced BVR-A levels or impaired BVR-A activation contribute to the development of brain insulin resistance and metabolic alterations in Alzheimer’s disease. In this narrative review, we will provide an overview on the literature by focusing on the role of BVR-A in the regulation of insulin signaling and how BVR-A alterations impact on cell dysfunctions in both metabolic and neurodegenerative disorders.

## 1. Introduction

Obesity and associated metabolic disorders, such as type 2 diabetes (T2D), metabolic syndrome, and non-alcoholic fatty liver disease (NAFLD), have taken an epidemic stature over recent decades [1,2,3,4]. Concurrently, the prevalence of neurodegenerative diseases is rising worldwide [5], and Alzheimer’s disease (AD) in particular represents a major and pressing health challenge with no treatment [6]. Considerable overlap has been identified in the risk factors, comorbidities, and pathophysiological mechanisms of obesity, T2D, and AD [7,8,9,10]. Much is known about the biology of these conditions, but whether they are parallel phenomena occurring from coincidental roots in ageing or synergistic diseases linked by vicious pathophysiological cycles remains unclear [11]. Experimental data suggested that therapies targeted at restoring metabolic homeostasis may improve cognitive function as well as increase lifespan in neurodegenerative diseases [12,13]. Likewise, uncontrolled, progressive weight gain and abnormal glucose tolerance are common metabolic dysfunctions observed in AD, which appear to negatively impact overall prognosis through an, as of yet, poorly defined series of mechanisms [14]. Insulin resistance is a core feature of metabolic disorders and growing evidence shows that it also contributes to AD pathogenesis [15,16,17,18]. Therefore, AD could be regarded as a metabolic disease mediated in part by brain insulin resistance. Even though many resources have been invested in attempting to elucidate and overcome insulin resistance, the molecular mechanisms at the base of such an alteration remain poorly understood [19]. Mapping the biochemical changes at the level of insulin signaling during metabolic and neurodegenerative disorders is becoming a priority, as filling knowledge gaps about disease mechanisms and their links can lead to developing much-needed therapies. The insulin signaling pathway contains several regulatory checkpoints, which represent critical nodes, and among them, biliverdin reductase-A (BVR-A) has recently emerged for its regulating functions [20].

Here, we review the growing literature concerning the role of BVR-A in metabolic and neurodegenerative diseases, aiming to highlight key observations and experimental data from studies conducted both in animal models and in humans, focused on the link between BVR-A dysregulation and insulin signaling alterations in metabolic disorders and neurodegenerative diseases.

## 2. BVR-A Is a Protein with Pleiotropic Nature

In 1965, Singleton JW described for the first time the biliverdin reductase (BVR) protein from guinea pig liver [21]. Later, Maines MD and colleagues purified and characterized BVR from rat liver [22], and in 1993 they described human BVR [23].

BVR has two isoforms with a different molecular weight: A and B. BVR-B is prevalent during fetal development, whereas BVR-A is ubiquitously expressed in adult tissues [24,25].

From a structural point of view, BVR-A is a monomeric protein that consists of two major regions, the catalytic and the regulatory/DNA interaction domains. The N terminus, the catalytic domain (Rossmann fold), contains a binding motif for nicotinamide adenine dinucleotide phosphate [NADP(H)] and nicotinamide adenine dinucleotide [NAD(H)] cofactors, which are used at different pH optima: 6.7 and 8.7, respectively. Being characterized by the dual pH/dual cofactor, this oxidoreductase is quite unique. This feature allows BVR-A to work in different cellular compartments and under diverse and adverse intracellular conditions [26,27]. Moreover, the C-terminal domain of BVR-A consists of a large, six-stranded b-sheet that hosts the bulk of key signaling sequences: the leucine zipper (bzip) motif, adenine dinucleotide-binding motif, serine/threonine kinase domain, Src homology (SH2) binding domains, and Zn/metal-binding motif [26,27].

**Figure 1:**
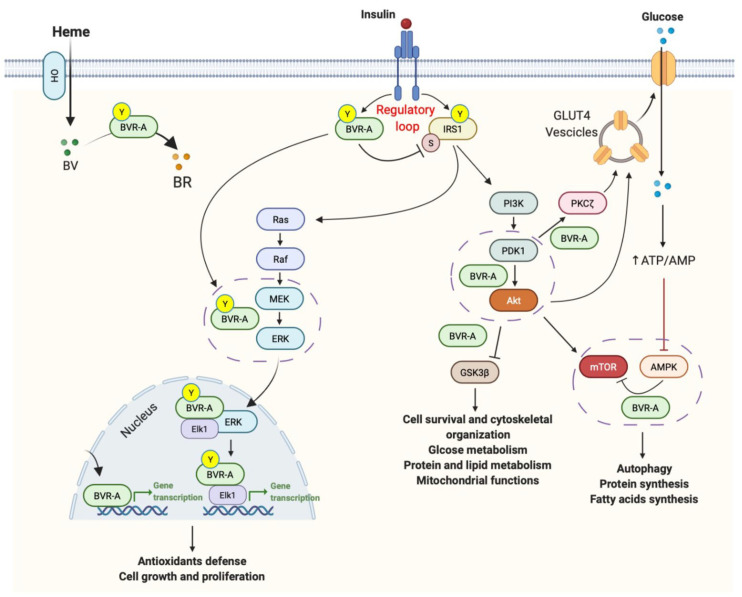
Known sites of BVR-A interaction in the insulin signaling pathway. Under physiological conditions, the activation of insulin signaling requires the binding of insulin to the insulin receptor (IR), which auto-phosphorylates on Tyr residues (Y, e.g., Tyr1158/1162/1163) and promotes the receptor tyrosine kinase-mediated phosphorylation of its substrate (IRS1) on specific Tyr residues (e.g., 632). In parallel, IR phosphorylates BVR-A on specific Tyr residues and activates BVR-A to function as Ser/Thr/Tyr kinase. Then, as part of a regulatory loop, BVR-A phosphorylates IRS1 on inhibitory Ser residues (S, e.g., Ser307) to avoid IRS1 aberrant activation in response to IR. Once activated, IRS1 works as a scaffold protein, driving the activation of the two main arms of the insulin signaling: (1) the Ras/Raf/MAPK pathway (ERK1/2) mainly involved in gene transcription; and (2) the PI3K/Akt axis that is critical for glucose uptake as well as for protein and lipid metabolism. Moreover, Akt promotes the phosphorylation of several targets, among which are: (1) GSK3β (on Ser9, inhibitory site), which has a role in energy production; and (2) mTOR (on Ser2448, activating site), which regulates protein synthesis and autophagy. Within both axes, BVR-A works as kinase or as scaffold protein facilitating: (1) ERK1/2 phosphorylation and the subsequent translocation in the nucleus and followed by the activation of Elk1; (2) the PDK1-mediated activation of Akt; (3) PDK1-mediated activation of the atypical PKCζ; (4) the Akt-mediated inhibition of GSK3β. Moreover, BVR-A was found to be essential for the AMPK-mediated inhibition of mTOR. Under condition of energy depletion, AMPK directly senses increases in AMP:ATP and ADP:ATP ratios, thus promoting the inhibition of mTOR to block the processes that deplete cellular ATP (e.g., protein synthesis and cell cycle progression, controlling cell size and preventing apoptosis). Moreover, AMPK is activated in response to nitrosative stress and that occurs independently of AMP/ATP levels. Conversely, reduced AMPK activation leads to mTOR hyper-activation. Arrows represent stimulation; lines represent inhibition. See text for more details.

From a functional point of view, BVR-A was traditionally known as the reductase that catalyzes the last step in the heme-degradation pathway, driving, in a powerful redox cycle, the conversion of biliverdin, a product of heme oxygenase (HO) activity, to bilirubin, the major physiological antioxidant [28,29,30] (Figure 1). Intriguingly, BVR-A also participates in cell signaling through several distinct tracks, and the range and diversity of its functions is unmatched by any enzyme characterized to date (reviewed in [25,26,27,31,32]). In particular, BVR-A has been demonstrated to be also endowed with a dual-specificity serine/threonine/tyrosine (Ser/Thr/Tyr) kinase activity [33,34], directly involved in the regulation of the complex insulin signaling pathway at different levels, influencing many metabolic processes, such as glucose uptake, regulation of lipid and protein metabolism, cell proliferation, differentiation, and death [20,35,36,37,38,39,40,41,42] (Figure 1).

Last but not least, being a bZip DNA binding protein, BVR-A can also act as a transcription factor for activator protein 1 (AP-1)- and cyclic adenosine monophosphate (cAMP)-regulated genes, modulating, among others, activating transcription factor-2 (ATF-2) and HO-1 expression, key components of the inflammatory and stress-responsive system [43,44,45,46,47,48,49,50] (Figure 1).

## 3. BVR-A in Insulin Signaling Pathway

The activation of insulin signaling promotes multiple effects, including: (a) the synthesis of proteins involved in glucose metabolism; (b) the activation of antiapoptotic mechanisms; as well as (c) the modulation of antioxidant defenses [51,52].

From a molecular point of view, insulin mediates the above-cited effects through two major pathways activated downstream from the insulin receptor (IR): the phosphoinositide 3-kinases (PI3K) pathway, mainly involved in metabolic signaling, and the mitogen-activated protein kinase (MAPK) pathway, which is associated with the transcriptional and mitogenic effects of insulin (Figure 1). The equilibrium between the strength of these two paths determines which downstream targets are preferred [51].

Interestingly, several data have shown a key role for BVR-A in the regulation of both pathways [20,35,36,37,38,39,40].

Insulin responses are promoted through the phosphorylation (activation) of the insulin receptor substrates-1 and -2 (IRS-1 and IRS-2) complexes. Such activation is achieved following the binding of insulin to the extracellular domain of the IR, which autophosphorylates on tyrosine (Tyr) residues. Such phosphorylation is required for the activation of IR kinase activity, which in turn promotes IRS1 phosphorylation. The coupling between IR and IRS1 is crucial for the induction of the intracellular cascade. Like IRS-1, BVR-A is a direct target of IR [20], which phosphorylates both BVR-A and IRS-1 on specific Tyr residues, thus resulting in their activation [20] (Figure 1). IRS-1 mediates the activation of the PI3K/protein kinase B (Akt), which favors the translocation of the glucose transporter type 4 (GLUT4) to the plasma membrane to mediate glucose uptake (Figure 1). In parallel, as part of a regulatory loop, BVR-A works by phosphorylating IRS-1 on Ser residues, i.e., Ser307 acting as inhibitory site, to avoid an excessive activation of IRS-1 in response to insulin [20]. In that way, BVR-A may be considered an upstream regulator of the whole insulin pathway (Figure 1). Furthermore, downstream from IRS1, BVR-A functions as scaffold protein, favoring: (i) the 3-phosphoinositide-dependent protein kinase 1 (PDK1)-mediated activation of Akt [22]; (ii) the PDK1-mediated activation of the atypical protein kinase C-ζ (aPKCζ) [38,53], known to regulate GLUT-4 translocation, and (iii) the Akt-mediated inhibition of the glycogen synthase kinase-3β (GSK-3β) [54] (Figure 1). Finally, BVR-A encompasses specific motifs in its sequence by which it modulates IR kinase activity either positively or negatively [35], and consequently regulates the degradation of the peroxisome proliferator activated receptor alpha (PPARα).

As we have mentioned above, the MAPK signaling is also influenced by BVR-A [23]. The MAPK family consists of three important groups: extracellular signal-regulated kinases (ERK), c-Jun N-terminal kinases (JNK), and p38. In particular, BVR-A is a nuclear transporter of the MAPK kinase (MEK)-activated ERK1/2. Hence, it takes part in the activation of the ETS Transcription Factor (Elk1), the transcription factor for oxidative-stress-responsive genes, i.e., HO-1 and inducible nitric oxide synthase (iNOS) [39,40] (Figure 1).

Moreover, data collected so far have identified BVR-A as a key modulator of members of conventional and atypical groups of protein kinase C (PKC) isozymes that link the two arms of the insulin pathway, the PI3K and MAPK pathway [37]. BVR-A and PKCs are activated by insulin, reactive oxygen species (ROS), and cytokines, including tumor necrosis factor-a (TNF-a) [38], and a few lines of evidence show that BVR-A stimulates PKCs autophosphorylation, has a function in the intracellular trafficking of PKCs, and enhances the cytokine-mediated nuclear factor kappa B (NF-kB)-promoter activity [38].

Considering the multidimensional input of BVR-A in the different insulin signaling pathways and the breadth of cell functions that can be potentially influenced, it is not surprising that in recent years researchers have focused on studying the role of this protein in the mechanisms of insulin resistance that underlie cognitive and metabolic deterioration in metabolic and neurodegenerative disorders.

## 4. Alteration of BVR-A in Metabolic Impairment: Data from Animal and Human Studies

### 4.1. Animal Studies

Several experimental data from animal studies support the role of BVR-A in regulating insulin signaling and participating in the maintenance of metabolic homeostasis.

In 2016, Gibbs PE and coworkers [55] demonstrated, in diabetic mice, that the stimulation of BVR-A kinase activity through a synthetic peptide ameliorated insulin signaling and increased glucose uptake, by the up-regulation of the IR/Akt/GSK3β axis [55] (Table 1).

In the same period, Hinds et al. [56] developed liver-specific BVR-A knock-out (KO) mice and found significantly higher liver weight, hepatic triglycerides, and Oil Red O staining in the liver after a high-fat diet (HFD) in the KO compared with the wild-type mice [56]. Moreover, the liver-specific BVR-A KO mice had upregulated de novo lipogenesis enzymes, including fatty acid synthase [56]. Liver-specific BVR-A KO mice also showed a decrease in the phosphorylated form of the AMP-activated protein kinase (AMPK), a downstream inhibitor of enzymes involved in fatty acid synthesis, and an increase in the active form of acetyl-CoA carboxylase [62], the rate-limiting enzyme in fatty acid synthesis (Table 1). These results suggest that BVR-A is an essential factor that mediates fat accumulation in the liver. In addition, in liver-specific BVR-A KO mice, there was an increase in GSK3β activity and a decrease in PPARα levels [56] (Table 1). As mentioned above, BVR-A plays a pivotal role in preventing the inhibition of PPARα by favoring the inhibitory phosphorylation of GSK3β, and this implies the upregulation of β-oxidation and the downregulation of de novo lipogenesis genes to promote lipid metabolism and glycogen storage in the liver [56].

The same research group [57] also generated an adipocyte-specific deletion of BVR-A in mice to determine the function of BVR-A in adipose tissue expansion. The KO and wild-type mice were placed on HFD for 12 weeks, and data showed that the percent body fat and body weights did not differ between the groups. However, KO mice had significantly higher visceral fat, with decreased mitochondrial number [57]. The BVR-A KO mice also had significantly higher fasting blood glucose levels and reduced levels of phosphorylated Akt and Glut4 mRNA [57] (Table 1). These results confirm the essential role of BVR-A not only in insulin signaling, but also in the inflammatory and stress response of adipose tissue. Intriguingly, Hinds TD Jr [36] recently demonstrated that rats genetically selected for high aerobic exercise capacity presented upregulated hepatic BVR-A [58].

While these results all point on a crucial role for BVR-A in the regulation of insulin signaling and related pathways, a very recent work from Stocker R and colleagues questioned the concept that a lack of BVR-A triggers insulin resistance [59]. This study shows that BVR-A KO mice respond to high fat diets comparably to wildtype littermate animals with regard to glucose metabolism and insulin sensitivity. Despite that, BVR-A KO mice challenged with high fat diets develop a fatty and inflamed liver [59] (Table 1). These observations certainly deserve further analyses.

### 4.2. Human Studies

Our group [60] evaluated, for the first time in humans, levels of BVR-A and its activation state as protein kinase in peripheral blood mononuclear cell (PBMC) from obese subjects and healthy controls to investigate the related molecular alterations in insulin signaling. We demonstrated that BVR-A levels were significantly reduced in obese subjects and this alteration was strictly associated with an aberrant activation of the IR/IRS1/Akt/GSK-3β/GLUT4 pathway [60] (Table 1). Our data also showed that BVR-A reduced levels in obese individuals strictly correlated with the presence of clinical features of metabolic syndrome [60]. In our study, the obese cohort underwent bariatric surgery for clinical purposes. Intra-operatory liver biopsy was performed for evaluating the presence of NAFLD, and visceral adipose tissue samples were collected for exploring the presence of local inflammation. Findings displayed that, in obese subjects, lower BVR-A protein levels were associated with the presence and severity of NAFLD and with adipose tissue dysfunction [60] (Table 1). Finally, it is interesting that in a sub-group of patients that were re-evaluated six months after bariatric surgery, we observed a recovery of BVR-A levels along with an improvement of insulin signaling [60].

Afterwards, Ceccarelli V et al. [61] conducted an immunohistochemical study focused on the analysis of BVR-A expression in omental adipose tissue from obese individuals and reported that, in this population, reduced BVR-A levels associated with a larger adipocyte size and greater expression of inflammatory and hypoxia markers [61] (Table 1).

In light of the fascinating results previously obtained, our group [63] recently studied BVR-A in T2D, observing that diabetic subjects had lower BVR-A protein levels compared to controls and that this alteration was associated with a more severe glyco-metabolic impairment and with an increased inflammatory condition [63] (Table 1).

The overall data suggest that BVR-A, regulating different molecular pathways of the insulin signalling pathway, plays a major role in the processes underlying the development of dysmetabolic conditions and may become an appealing target for novel therapeutic approaches.

## 5. BVR-A in Brain Insulin Resistance and Neurodegenerative Disorders

The brain represents only the 2% of the body’s total weight, although it is characterized by a high demand for energy compared to other tissues. Such a great request can be largely attributable to the extremely active and complex processes involved in neuronal transmission [64]. Neuronal cells are a paramount example of extraordinary energy expenditure for their functions and survival. This situation is mirrored in the huge metabolic rates in neurons as well as in the relatively higher susceptibility of brain tissues to oxygen and glucose deprivation. Reactions governing the conversion of nutrients into available cytosolic levels of adenosine triphosphate (ATP) are critical to generate the potential metabolic work that is available to a neuron at any given time [65,66].

To note, about 70% of the total energy is consumed for regulating neuronal signaling (resting and action potentials, postsynaptic receptor signaling, the glutamine cycle, and postsynaptic Ca2+), and only 30% is consumed for non-signaling activities (proteins, phospholipids, etc.). Indeed, the inability to preserve basal energy levels can result in synaptic dysfunctions and cognitive impairment, thus rendering the brain highly exposed to energy deficit-mediated injury [62,67].

Glucose is an essential substrate for the adult brain [68,69,70]. Approximatively 25% of glucose uptaken within the brain is conveyed into metabolic processes responsible for energy production required to drive basal brain activities [68,69,70]. While the majority of glucose uptake in neurons occurs through the glucose transporter 3 (GLUT3), insulin-regulated GLUT4 is also co-expressed with GLUT3 in brain areas regulating cognitive behaviors [17]. These regions include the basal forebrain, hippocampus, amygdala and, to lesser degrees, the cerebral cortex and cerebellum. Under physiological conditions, insulin induces GLUT4 translocation to the neuronal cell membrane via an Akt-dependent mechanism [17] and is believed to increase glucose flux within the neurons particularly during periods of high metabolic demand, i.e., learning and memory [17].

So far, a growing number of studies support the idea that the brain is an insulin-sensitive organ [17,18]. IR is broadly distributed throughout the brain, including areas regulating autonomic, emotional, and cognitive functions [17,18]. Indeed, besides mediating glucose uptake, activation of the insulin signalling in the brain plays a pivotal role in the regulation of hippocampal plasticity as well as learning and memory functions [17,18].

Conversely, elevated levels of insulin resistance markers within the brain are associated with worse performance on cognitive tests of episodic and working memory, suggesting a role for insulin signaling in neuronal functions [71,72,73]. From a molecular point of view, these dysfunctions might manifest as the impairment of neuroplasticity, receptor regulation, or neurotransmitter release in neurons [74,75,76,77,78]. In addition, dysfunctions of processes involved in insulin metabolism, such as neuronal glucose uptake in neurons expressing GLUT4, or homeostatic or inflammatory responses to insulin [77,78,79,80,81,82], can be observed.

Consistent with the role for BVR-A in regulating insulin signalling, several studies have highlighted the association between impaired BVR-A and brain insulin resistance development. Most of these research works mainly focus on AD pathology, AD being a neurodegenerative disease extensively studied to understand the role of brain insulin resistance on its pathogenesis.

### 5.1. Animal Studies

The first work highlighting a role for BVR-A in the development of brain insulin resistance was published in 2016 [81]. Data collected in a longitudinal study showed that the impairment of BVR-A is one of the earliest events observed during the development of brain insulin resistance in 3xTg-AD mice (a model to study AD) and that this phenomenon occurs before a consistent accumulation of Aβ and Tau pathology (well-known AD markers [83]) as well as increased TNFa levels (a main inducer of insulin resistance [79,80]) in the brain [81] (Table 2). In particular, a role for oxidative and nitrosative stress in mediating BVR-A dysfunction was proposed, whereby the observed increase of oxidative and nitrosative stress in the hippocampus of 3xTg-AD mice were responsible for the oxidative damage observed at the expense of BVR-A, named the increase of 3-nitrotyrosine (3-NT) [81] (Table 2). Similar results were collected in neuronal cells in vitro exposed to hydrogen peroxide (H_2_O_2_) or peroxynitrate (ONOO-) [81]. Notably, these events were antecedent to the frank inhibition of IRS1 in the brain [81]. BVR-A nitration impairs its Ser/Thr/Tyr kinase activity, which resulted initially in the hyper-activation of IRS1 (in agreement with the regulatory role for BVR-A discussed above) and later in the inhibition of IRS1 mediated by the activation of inhibitory feedback mechanisms, such as the hyper-activation of the mammalian target of rapamycin (mTOR) [81].

Subsequently, in another study, our group showed that the dysfunction of BVR-A have produced harmful effects downstream from IRS1. Indeed, despite IRS1 hyper-activation, neither an augmented activation of Akt nor a greater inhibition of GSK-3β in the hippocampus of 3xTg-AD mice, were observed [73] (Table 2). Remarkably, lack of BVR-A reduced the physical interaction between Akt and GSK-3β [73] (Table 2). These data are in agreement with the role for BVR-A in favoring the PDK1-mediated activation of Akt as well as the Akt-mediated inhibition of GSK-3β in response to insulin [36,54]. These alterations were associated with a significant impairment of cognitive and learning functions in 3xTg-AD mice [73,84,85,86], and of long-term potentiation (LTP) evaluated in cortical neurons [75] (Table 2). Therefore, oxidative stress-induced damage of BVR-A may turn-off the activation of insulin signaling, diminishing its neuroprotective effects before a frank brain insulin resistance develops.

**Figure 2:**
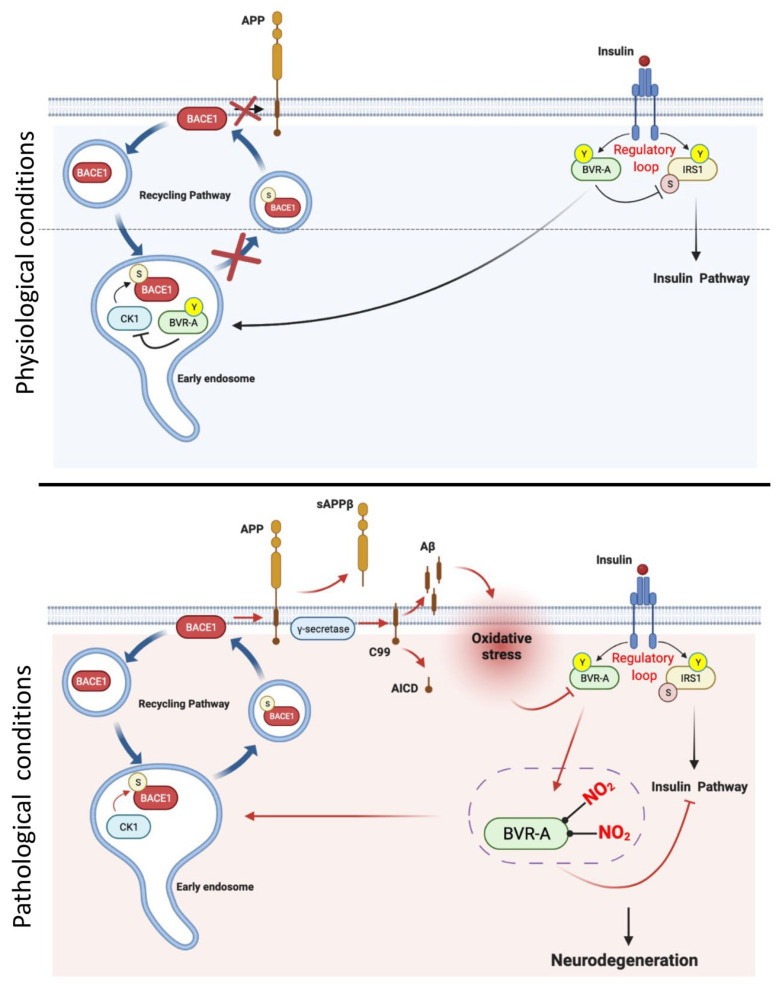
Hypothesized mechanism through which impairment of BVR-A links brain insulin resistance with increased Aβ production in Alzheimer disease (AD). Under physiological conditions insulin receptor (IR) phosphorylates biliverdin reductase-A (BVR-A) on tyrosine (Y) residues promoting BVR-A kinase activity and scaffold functions. Through these activities BVR-A regulates the activation of insulin signalling. Moreover, BVR-A activation plays a critical role in the inhibition of beta secretase 1 (BACE1) recycling to the plasma membrane. The proposed path probably occurs at the level of the early endosomes where casein kinase 1(CK1)-mediated Ser phosphorylation of BACE1 favors BACE1 recycling to plasma membrane. Under physiological conditions, BVR-A inhibits CK1 activity, thus preventing the phosphorylation of BACE1 and the subsequent recycling at plasma membrane. Conversely, during the progression of Alzheimer disease (AD) pathology the increase of oxidative stress leads to reduced BVR-A Tyr phosphorylation and increased BVR-A nitration (3-NT) that (1) impairs the activation of the insulin signalling pathway and in parallel (2) promotes CK1-mediated BACE1 recycling at the plasma membrane, where BACE1 cleaves amyloid precursor protein (APP), leading to increased beta amyloid (Aβ) production. In turn, as in a vicious cycle, increased Aβ oligomers trigger the elevation of oxidative and nitrosative stress levels, which further impair BVR-A. Arrows: activation; lines: inhibition. Red arrows/lines, molecular pathways activated during AD.

Brain insulin resistance was proposed to accelerate the production and accumulation of beta amyloid (Aβ) in an AD brain [73,84,85,86]. Consequently, as in a vicious cycle, increased Aβ oligomers generation further exacerbate brain insulin resistance [87]. Our group uncovered an original mechanism linking BVR-A dysfunction, brain insulin resistance and increased Aβ production [82] (Table 2). In a study performed in aged beagles (a natural higher mammalian model of aging/AD), we found that BVR-A undergoes similar modifications to those observed in AD mouse models that were associated with markers of brain insulin resistance [87] (Table 2). Specifically, we proposed that IR-mediated Tyr phosphorylation of BVR-A is required for preventing beta secretase 1 (BACE1) recycling to the plasma membrane and thus avoiding the amyloidogenic cleavage of the amyloid precursor protein (APP) (Figure 2). This pathway likely occurs at the level of the early endosomes where casein kinase 1(CK1)-mediated Ser phosphorylation of BACE1 triggersBACE1 recycling to plasma membrane [88,89]. We therefore hypothesized that during the progression of AD, the early dysfunction of BVR-A (1) impairs the activation of the insulin signaling pathway and in parallel (2) fosters CK1-mediated BACE1 recycling at the plasma membrane, where BACE1 cleaves APP, increasing Aβ production. In turn, as in a vicious cycle, Aβ oligomers may prompt the increase of oxidative and nitrosative stress levels, further impairing BVR-A [82] (Figure 2). These results highlight a role for BVR-A as molecular target whose dysfunction links brain insulin resistance, increased Aβ production, and elevated oxidative stress levels in AD.

Increased 3-NT modifications at the expense of BVR-A were also associated with mTOR hyper-activation [75,81,82] (Table 2). mTOR is a master regulator of the autophagic process [90]. The hyper-activation of mTOR, which negatively regulates autophagy induction, along with a reduced autophagic flux was reported in AD and AD-related models [91,92,93,94,95,96]. Moreover, mTOR hyper-activation is also among the known feedback mechanisms promoting IRS1 inhibition and insulin resistance development in AD [91,92,93,97]. Our group further showed that BVR-A impairment is a critical event prompting the disruption of the AMPK/mTOR axis and resulting in mTOR hyper-activation in the brain [98] (Table 2). This aspect is quite fascinating considering the role of AMPK as a key cellular energy sensor and as regulator of metabolic processes, including those stimulated by insulin [99]. Indeed, AMPK was found to be dysregulated in metabolic disorders, i.e., diabetes, obesity, as well as in neurodegenerative diseases [100,101]. Hence, a dysregulation of the BVR-A/AMPK axis may have a role in mTOR-mediated brain insulin resistance development (Figure 1).

Among the strategies to improve insulin signaling activation in the brain, the effects of intranasal insulin (INI) administration are being evaluated in an ongoing trial in the field of AD. Intranasal administration is a conceivable strategy to bypass the blood–brain barrier, allowing drugs to directly reach the brain, thereby avoiding side effects caused by systemic administration [102]. INI improved memory and attention in healthy participants [103] and in both mild cognitive impairment (MCI) and AD subjects [103,104,105] as reported in small-scale clinical trials. Moreover, preclinical studies performed in mouse models of aging or AD confirmed the beneficial effects of INI on cognitive functions [106,107,108,109,110] and highlighted the path by which insulin reaches the brain [111,112,113].

To further support the hypothesis that BVR-A has a role in insulin signaling, the effects of INI administration on BVR-A and insulin signalling activation were evaluated in the brain of both adult (six months of age) and aged (12 months of age) 3 × Tg-AD mice [75] (Table 2). Collected results showed that INI administration (1) prevented the early impairment of BVR-A in adult mice and (2) retrieved BVR-A activation in aged mice (Table 2). The rescue of BVR-A activation was positively associated with the amelioration of the insulin signaling pathway in the brain along with an improvement of AD neuropathology, cognitive, and non-cognitive functions [75] (Table 2). Within this study, in vitro experiments also highlighted that a lack of BVR-A functions is detrimental for cells and drives them toward insulin resistance, while recovering BVR-A activities rescue insulin signalling activation [75]. These results further support the hypothesis that the impairment of BVR-A is an early event along the development of brain insulin resistance. Hence, BVR-A likely represents a novel therapeutic target to prevent/rescue brain insulin signaling alterations in AD.

Finally, a recent study published in 2021 highlighted that the impairment of BVR-A may trigger the development of brain insulin resistance also in a mouse model for Down syndrome (DS), thus representing a shared mechanism between DS and AD [78] (Table 2). DS is a genetic disorder caused by trisomy of chromosome 21 and it is currently considered a genetic model of accelerated aging and early-onset, genetic AD [114,115,116]. Age-dependent reduction of BVR-A Tyr phosphorylation was observed in frontal cortex samples isolated from Ts65dn mice compared to euploids and this phenomenon was associated with increased IRS1 inhibition and mTOR hyper-activation, supporting a role for dysfunctional BVR-A in favoring brain insulin resistance development in DS [78].

### 5.2. Human Studies

The first observation highlighting a role for BVR-A in the brain was published in 2011, by the Butterfield’s group [117,118]. They showed that alterations in terms of BVR-A activation were characterized by decreased Tyr phosphorylation and increased oxidative/nitrosative post-translational modifications in the brain of both MCI and AD subjects [117,118] (Table 2). These data further pointed to an aspect often ignored in earlier studies, namely that increased BVR-A protein levels are not always associated with increased phosphorylation/activation. Indeed, total BVR-A protein levels were found to be increased while BVR-A Tyr phosphorylation was significantly reduced in MCI and AD subjects thus suggesting an overall impairment of BVR-A activity [117,118]. Reduced BVR-A Tyr phosphorylation may be due to the consistent nitrosative stress-induced modifications at the expenses of BVR-A, i.e., the 3-NT modifications observed both in AD and MCI [117,118] (Table 2). Because oxidative/nitrosative post-translational modifications modify protein structure [119] and often result in reduced functions [120] it is conceivable to think that increased 3-NT levels on BVR-A might be responsible for reduced Tyr phosphorylation and thus BVR-A activation. To note, nitration and phosphorylation processes compete for same residues, i.e., Tyr residues [121]. In particular, from a chemical point of view, steric hindrance of the NO_2_ group on the 3-position of Tyr could negatively impact on Tyr kinases activity for the 4-OH group. This notion reinforces the hypothesis that nitrosative stress prevents/inhibits Tyr phosphorylation on BVR-A [117,118].

Interestingly, the evaluation of the insulin signalling pathway performed on the same samples in a subsequent study [94] revealed that increased IRS1 inhibition (a marker of insulin resistance) and mTOR hyper-activation can be observed in both aMCI and AD post-mortem hippocampal samples, previously shown to be characterized by BVR-A impairment [117,118] (Table 2). These observations further support the role for BVR-A in regulating insulin signalling also in humans.

Indeed, as observed in animal models, the impairment of BVR-A also significantly impacts its scaffold activity downstream from IRS1. In a study published in 2019, Sharma et al. reported that reduced BVR-A protein levels impair the physical interaction between Akt and GSK-3β in aMCI and AD post-mortem cortical samples, thus reducing the Akt-mediated inhibition of GSK-3β [54] (Table 2). Notably, the loss of BVR-A may impair the Akt-mediated inhibition of GSK-3β through at least two mechanisms: (1) by precluding the activation of Akt (consistent with previous data from Maines’ group [36]); and (2) by avoiding the physical interaction between Akt and GSK-3β [54]. In that way, the impairment of BVR-A activity precludes the insulin signalling activation downstream from IRS1.

In addition, in aMCI and AD post-mortem brain samples, BVR-A dysfunction negatively impacts on survival pathways activated in response to insulin, such as the MAPK signalling, thus further worsening AD neuropathology. Indeed, phosphorylated BVR-A is a scaffold protein participating in the activation of ERK1/2 by MEK1/2 and then of Elk1 by ERK1/2 [39] (Table 2). Hence, reduced BVR-A phosphorylation along with the decreased interaction with ERK2 observed in an AD hippocampus [117] lend support to the hypothesis that BVR-A may contribute to the observed ERK1/2 dysfunction in AD [122,123,124,125].

Based on the results cited above and collected in mice [78], we can also hypothesize a role for impaired BVR-A in triggering brain insulin resistance and AD in DS. Indeed, an increased BVR-A nitration along with reduced BVR-A activation were reported in post-mortem frontal cortex samples from DS individuals who developed AD [126] (Table 2). This evidence is consistent with the increased IRS1 inhibition found in the same samples [72], thus reinforcing the idea that the dysfunction of BVR-A may be a shared mechanism among different pathologies.

**Table 2:** Summary of the studies highlighting a link between BVR-A alterations and insulin signaling-related pathways in neurodegenerative diseases.

Model	BVR-A Alteration(s)	Insulin Signaling Alteration(s)	Observed Effect(s)	Ref.
3xTg-AD mice	Reduced BVR-A levels and activation (6–18 months) and increased 3-NT on BVR-A (12–18 months) in the hippocampus	IRS1 hyper-activation (6 months) followed by increased IRS1 inhibition and mTOR hyper-activation (12 months)	Increased Aβ and Tau phosphorylation in the hippocampus	[81]
3xTg-AD mice	Reduced BVR-A levels and Tyr-phosphorylation (6–12 months) in the hippocampus and cortex	IRS1 hyper-activation (6 months) followed by increased IRS1 inhibition (12 months); reduced Akt activation and reduced GSK3β inhibition on Ser9 (6–12 months); reduced Akt-GSK3β physical interaction (6–12 months); ERK1/2 hyperactivation (6 months); mTOR hyper-activation (12 months)	Impairment of cognitive and learning functions (6–12 months); increased Aβ levels and Tau phosphorylation in the hippocampus and cortex (6–12 months)	[73,75]
3xTg-AD mice treated with intranasal insulin	Increased BVR-A Tyr-phosphorylation in the hippocampus and conrtex (6 and 12 months)	Reduced IRS1 hyper-activation (6 months) and reduced IRS1 inhibition (12 months); increased Akt activation (6 and 12 months); block of mTOR hyper-activation (12 months)	Improvement of cognitive and learning functions (6 and 12 months); reduced Aβ levels and Tau phosphorylation in the hippocampus and cortex (6 and 12 months)	[75]
C57Bl6 mice	Reduced BVR-A levels and phosphorylation (12 months) and increased 3-NT on BVR-A (18 months) in the hippocampus	Increased IRS1 inhibition (18 months)	-	[81]
Canine (beagle)	Reduced BVR-A Tyr-phosphorylation (4–12 months) and increased 3-NT on BVR-A (10–12 months) in the parietal cortex	Reduced Akt activation (4–12 months)	Increased Aβ levels in the cortex (10–12 months)	[82]
BVR-A KO mice	Global BVR-A deficiency in the cerebral cortex	mTOR hyper-activation and reduced AMPK levels	Impairment of autophagic flux in the cortex	[98]
Ts65dn mice	Reduced BVR-A Tyr-phosphorylation in the frontal cortex (9 months)	Increased IRS1 inhibition; mTOR hyper-activation	Loss of proteins regulating synaptic plasticity; accumulation of APP-C99	[78]
aMCI and AD subjects	Reduced BVR-A Tyr-phosphorylation and increased 3-NT on BVR-A in the hippocampus	Increased IRS1 inhibition; mTOR hyper-activation; decreased interaction with ERK2	-	[94,117,118]
aMCI and AD subjects	Reduced BVR-A levels in the parietal cortex	Reduced GSK3β inhibition; reduced Akt-GSK3β physical interaction	-	[54]
DS subjects	Increased 3-NT on BVR-A and reduced BVR-A activation in the frontal cortex	Increased IRS1 inhibition; mTOR hyper-activation	-	[72,125]
Centenarians	Increased BVR-A gene expression in blood samples	-	-	[126]

Abbreviations: 3-NT: 3-nitrotyrosine; Aβ: beta amyloid; AD: Alzheimer’s disease; aMCI: aminestic mild cognitive impairment; AMPK: 5′ AMP-activated protein kinase; Akt: protein kinase B; APP-C99: amyloid precursor protein C-terminal fragment 99; BVR-A: biliverdin reductase-A; DS: down syndrome; ERK1/2: extracellular signal-regulated kinase 1/2; GSK3β: glycogen synthase kinase 3 beta; IRS1: insulin receptor substrate isoform 1; mTOR: mammalian target of rapamycin; Tyr: Tyrosine.

Finally, it is interesting to report that a very recent study conducted in centenarians found increased BVR-A gene expression in blood collected from centenarians compared to controls [127] (Table 2). The comprehension of the mechanisms associated with healthy aging is of great importance because aging is inexorably linked to the cumulative burden of age-associated diseases, such as cardiovascular disease (CVDs), stroke, type 2 diabetes, hypertension, different types of cancer, or dementia [128,129]. Results concerning the BVR-A gene coupled with the findings that most longevity-associated, rare coding variants converge upon conserved insulin/insulin growth factor-1 (IGF-1) signaling and AMPK signaling pathways [130] led us to hypothesize a role for BVR-A in the maintenance of insulin signalling in healthy aging and studies on this topic are ongoing in our lab.

## 6. Conclusions

The spread of a Western diet and lifestyle worldwide and the ever-increasing life expectancy has led to a dramatic rise in the prevalence of metabolic and age-associated disorders, which currently represent urgent global health challenges.

Significant overlap has been identified in the pathophysiological mechanisms of metabolic diseases, such as T2D, neurodegenerative disorders, and AD, and in particular the alteration of insulin signaling is a shared hallmark of these conditions, still not fully understood from a molecular point of view. Thus, better knowledge of the intersected molecular processes leading to the development of these conditions, we posit, may point to the bases to identify novel, necessary therapeutic targets.

In this context, most of the research conducted so far mainly focused on the role of IRS1 in the impaired activation of insulin pathway, while the fascinating role of BVR-A in the early alterations of the insulin pathway are still incompletely described and deserve deeper investigations.

**Figure 3:**
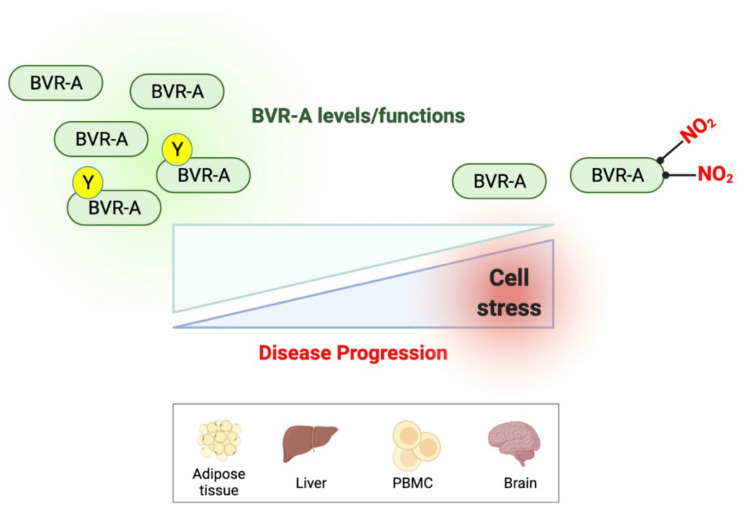
Reduced BVR-A protein levels or impaired BVR-A activation occur with the progression of metabolic or neurodegenerative diseases. Reduced BVR-A protein levels or impaired BVR-A activation (reduced Tyr phosphorylation or increased nitration) are observed during the progression of either metabolic or neurodegenerative diseases and are associated with the dysfunction of insulin signaling.

As emerged from studies performed in both human and animal models of obesity and neurodegenerative diseases, an impairment of BVR-A occurs before IRS1 inhibition and is associated with a reduced insulin signaling activation. Hence, reduced BVR-A protein levels or activation may conceivably represent an early marker for insulin resistance (Figure 3).

Looking to the future, deciphering a novel biological mechanism based on the role of the BVR-A protein to identify early alterations of the insulin signaling pathway may have a significant impact on both systemic and brain insulin resistance development. In fact, it is plausible to think that preventing BVR-A dysfunction or restoring BVR-A activities could represent a promising strategy to slow or stop the progression of insulin resistance, finally resulting in an amelioration of both metabolic and neurodegenerative disorders.

## Figures and Tables

**Table 1:** Summary of the studies highlighting a link between BVR-A alterations and insulin signaling-related pathways in metabolic diseases.

Model	BVR-A Alteration(s)	Insulin Signaling Alteration(s)	Observed Effect(s)	Ref.
Ob/Ob mice	Stimulation of BVR-A kinase activity by KYCCSRK peptide	Increase of IR activation	Rapid glucose clearance from the circulation	[55]
Liver-specific BVRA KO mice	Liver deletion of BVR-A	Reduced GSK3β inhibition	Impaired glucose tolerance and development of fatty liver	[56]
HFD-treated BVRA KO mice	Adipocyte deletion of BVR-A	Decreased Akt activation and reduced GLUT4 levels	High fasting blood glucose levels; adipocytes hypertrophy and reduction of mitochondrial number in white adipose tissue	[57]
BVRA KO mice	Global BVRA deficiency	-	Fatty liver without alteration in glucose metabolism and insulin sensitivity	[58]
Obese subjects	Reduced BVR-A levels in PBMC	Aberrant activation of insulin signalling characterized by: reduced IRS^Ser307^/IRS1 ratio; increased pAkt^Ser473^/Akt and increased pGSK3β^Ser9^/GSK3β ratio; increased AS160-mediated GLUT4 translocation	Metabolic syndrome, presence and severity of NAFLD and adipose tissue dysfunction	[59]
Obese subjects	Reduced BVR-A expression in visceral adipose tissue	-	Larger adipocytes size and greater local expression of inflammatory and hypoxia markers	[60]
T2D subjects	Reduced BVR-A levels in PBMC	-	Glyco-metabolic impairment and increased inflammatory condition	[61]

Abbreviations: BVR-A: Biliverdin Reductase A; Ob: obese; IR: Insulin Receptor; KO: Knock-out; GSK3β: glycogen synthase kinase 3 beta; HFD: high fat diet; GLUT4: glucose transporter type 4; PBMC: peripheral blood mononuclear cell; IRS: insulin receptor substrate; NAFLD: non-alcoholic fatty liver disease; T2D: type 2 diabetes.

## Data Availability

Not applicable.
